# Overexpression of collagen VI α3 in gastric cancer

**DOI:** 10.3892/ol.2014.1910

**Published:** 2014-02-25

**Authors:** XIAOJUN XIE, XIAOSUN LIU, QING ZHANG, JIREN YU

**Affiliations:** Department of Gastrointestinal Surgery, The First Affiliated Hospital, School of Medicine, Zhejiang University, Hangzhou, Zhejiang 310003, P.R. China

**Keywords:** collagen VI α3, microarray, meta-analysis, gastric cancer

## Abstract

Collagen VI is significant in the progression of numerous types of cancer. Type VI collagen consists of three α-chains and collagen VI α3 (COL6A3) encodes the α3 chain. The overexpression of COL6A3 has been demonstrated to correlate with high-grade ovarian cancer and contributes to cisplatin resistance; however, its role in human gastric cancer (GC) remains unclear. Using microarray meta-analysis, COL6A3 was observed to be frequently overexpressed in the GC tissues, furthermore, this overexpression was identified in five GC cell lines. A microarray-based co-expression network analysis was conducted and identified a total of 62 genes that were co-expressed with COL6A3, with the majority of the genes being involved in cancer-related processes, such as cell differentiation, migration and adhesion. Network analysis of these 62 genes demonstrated that fibronectin 1, a well-characterized oncogene, was located at the center of the COL6A3 co-expression network. Therefore, COL6A3 may act as an oncogene in human GC and the antagonism of COL6A3 may be an effective therapeutic treatment for GC.

## Introduction

Gastric cancer (GC) is the fourth most common type of malignancy worldwide, which results in 989,600 novel cases and 738,000 fatalities annually, specifically in Asian countries (1). Recent advancements in diagnosis and treatment modalities have been made, however, the prognosis of GC patients remains poor. As current therapeutic strategies are insufficient and do not achieve complete tumor ablation, it is important to analyze the molecular mechanisms of GC and identify novel biomarkers, as well as targets for therapeutic approaches, which may improve the clinical outcome for GC patients.

Collagen VI was initially identified as an extracellular matrix protein. It forms a microfilament network and binds to extracellular matrix proteins via its functional subdomains, which is important for the organization of fibrillar collagens and adhesion to the basement membrane (2). Collagen VI has recently attracted interest due to its involvement in breast and ovarian cancers (3–5). It is composed of three distinct α-chains (α1, -2 and -3) and collagen VI α3 (COL6A3) encodes the α3 chain, which is markedly longer than the other two chains (6). In a previous study, COL6A3 was shown to be upregulated in ovarian cancer (7), and Sherman-Baust *et al* (5) identified that the expression of COL6A3 was correlated with cisplatin resistance in ovarian cancer cell lines. Furthermore, highly or moderately differentiated ovarian tumors expressed lower levels of COL6A3 than poorly differentiated tumors, which indicated that the expression of COL6A3 was associated with the grade of the ovarian tumor (5). A recent exon array analysis study demonstrated that an alternative long isoform of COL6A3 was expressed, almost exclusively, in cancer samples, and may potentially serve as a novel cancer biomarker (8). Currently, the majority of studies relating to the oncogenic role of this gene focus on ovarian and breast cancer, however, the expression pattern and the biological functions of COL6A3 in human GC remain unknown.

In the present study, the authors investigated whether the expression level of COL6A3 was altered in GC, and a microarray meta-analysis was performed in order to assess the functional characteristics and molecular mechanisms of COL6A3 in GC.

## Materials and methods

### Gene expression patterns in GC

The Oncomine database (http://www.oncomine.org) was used to examine the differences in the transcriptional profiles between GC tissues and the adjacent normal tissues (9). Only the datasets that contained cancer versus normal analysis at the mRNA expression level were selected for analysis in the present study. In total, four GeneChip datasets, consisting of 318 paired GC and non-cancerous tissues, were selected according to the criteria shown in Table I.

### Cell culture

Five human GC cell lines (AGS, HGC-27, BGC-823, SGC-7901 and MGC80-3) and one immortalized gastric cell line (GES-1) were purchased from Shanghai Institute of Cell Biology (Shanghai, China). All cell lines were incubated in Dulbecco’s modified Eagle’s medium (Gibco-BRL, Carlsbad, CA, USA) with 10% fetal bovine serum (SAFC Biosciences Inc., Lenexa, KS, USA), 100 U/ml penicillin and 100 mg/ml streptomycin (Sigma-Aldrich, St. Louis, MO, USA).

### Quantitative polymerase chain reaction (qPCR) analysis

TRIzol reagent (Invitrogen Life Technologies, Carlsbad, CA, USA) was used to extract the total RNA from whole cells, and reverse-transcription was conducted using a TaqMan^®^ Reverse Transcription kit (Applied Biosystems, Foster City, CA, USA). The DNA was amplified using an ABI^®^ 7500 Real-Time PCR system (Applied Biosystems) and SYBR Premix Ex Taq (Takara, Kusatsu, Japan). The ΔΔCt method was used to calculate the relative RNA expression, which was normalized to GAPDH expression. PCR was performed using the following primers: forward, 5′-GAGACGCAGTGAGTGGGAAA-3′ and reverse, 5′-AGAGTCTTGTGCTGCTTGCT-3′ for COL6A3; and forward, 5′-CTCTCTGCTCCTCCTGTTCGAC-3′ and reverse, 5′-TGAGCGATGTGGCTCGGCT-3′ for GAPDH.

### Co-expression analysis

The Oncomine database co-expression analysis tool was used to conduct the co-expression analysis of the microarray datasets. Using the co-expression score, the top 150 genes of each dataset were selected. The genes that appeared in at least two of the three datasets were defined as COL6A3 co-expressed genes.

### Gene ontology (GO) and pathway enrichment analysis

GO and pathway enrichment analysis were conducted to examine COL6A3 co-expressed genes using the Database for Annotation, Visualization and Integrated Discovery (DAVID; http://david.abcc.ncifcrf.gov/). The categories, GOTERM_BP_3, GOTERM_CC_2 and GOTERM_MF_3 were selected, and the other options were set as defaults.

### Construction of the gene interaction network

The gene interaction network was constructed using a gene expression pattern scanner (GePS: http://www.genomatix.de/) as described previously (10).

### Statistical analysis

The independent Student’s t test was used to analyze the differences between two groups. Statistical analysis was performed using SPSS software version 16.0 (SPSS, Chicago, IL, USA). Data are presented as the means ± SD. P<0.05 was considered to indicate a statistically significant difference.

## Results

### COL6A3 is commonly overexpressed in GC

**Figure 1:**
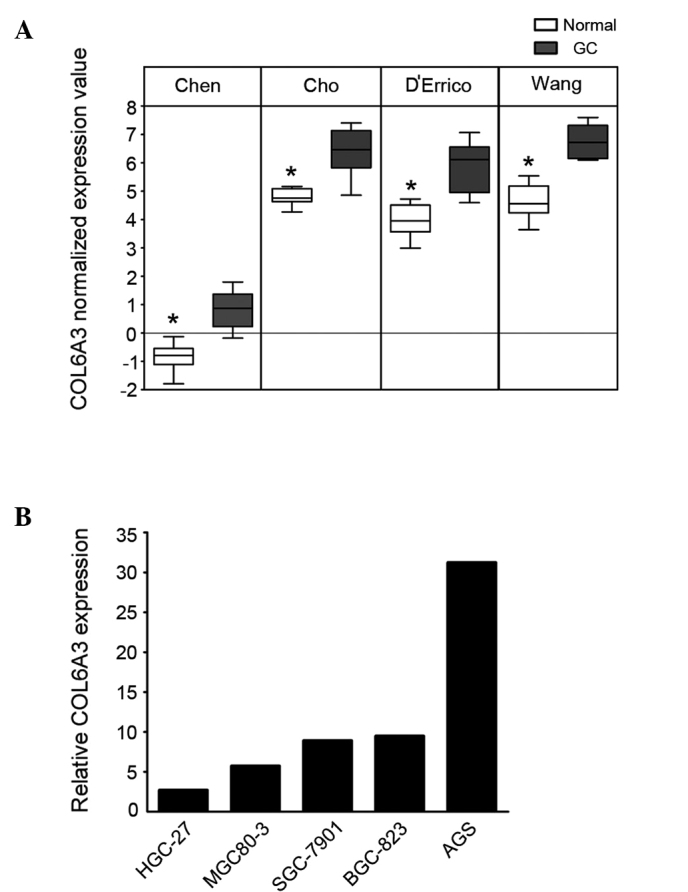
COL6A3 was overexpressed in gastric carcinoma tissue. (A) The expression pattern of COL6A3 in four GC datasets that were obtained using the Oncomine database; whiskers, 10th and 90th percentile; box boundaries, 75th and 25th percentile; line within the box, median. *P<0.001. (B) Relative COL6A3 expression of five GC cell lines (HGC-27, MGC80-3, SGC-7901, BGC-823 and AGS) compared with the mean value of a normal GC cell line (GES-1). COL6A3; collagen VI α3; GC, gastric cancer.

To determine the changes in the transcriptional pattern of GC cells, microarray datasets from the studies by Chen *et al* (11), Cho *et al* (12), D’Errico *et al* (13) and Wang *et al* (14) were analyzed using the Oncomine database. COL6A3 demonstrated a significant overexpression in the GC cells (P=3.98×10^−15^; Fig. 1A). To confirm this finding, the expression of COL6A3 in one immortalized gastric cell line (GES-1) and five GC cell lines (AGS, HGC-27, BGC-823, SGC-7901, MGC80-3) was analyzed using qPCR. The five GC cell lines exhibited ≥2.5-fold overexpression of COL6A3 compared with that of GES-1 cells (Fig. 1B).

### Genes co-expressed with COL6A3

A previous study indicated that genes which are co-expressed in different conditions may be functionally related or co-regulated (15). Therefore, a microarray co-expression analysis was conducted to identify the genes that were co-expressed with COL6A3. The dataset from the study by D’Errico *et al* (13) did not contain any co-expression data, therefore, the other three datasets consisting of 249 paired tissues were selected for inclusion in the co-expression analysis. Using a cut-off of the top 150 genes, which were identified by the co-expression score from each dataset, and with at least two appearances on the co-expressed list, 62 genes were identified as genes that were co-expressed with COL6A3 (Table II).

### GO and pathway enrichment analysis of COL6A3 co-expressed genes

**Figure 2:**
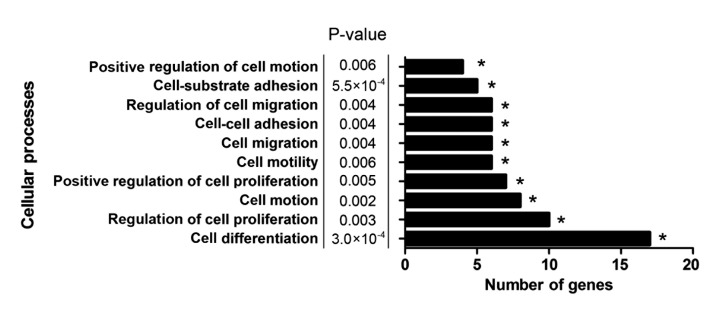
Gene ontology analysis of collagen VI α3 co-expressed genes was conducted using the Database for Annotation, Visualization and Integrated Discovery functional annotation chart tool. ^*^P<0.01 for the pathway enrichment of COL6A3 co-expressed genes compared with *Homo sapiens* transcriptome background.

GO and pathway enrichment analysis were conducted using the DAVID functional annotation chart tool (16) to further analyze the underlying mechanisms of COL6A3 and its co-expressed genes. In total, 36 biological process, seven cellular constituents, seven molecular function terms and six Kyoto encyclopedia of genes and genomes pathways were indicated to be significantly enriched (P<0.01; Table III). The extracellular matrix organization indicated the most marked enrichment among the GO biological process terms. The predominant function of COL6A3 has been identified to be the organization of matrix components, which supported the reliability of the present analysis. Furthermore, cell processes, such as cell differentiation, cell-substrate adhesion, regulation of cell proliferation, regulation of cell migration, cell motion and cell migration, which are considered to be cancer-related biological processes, were enriched (Fig. 2). This result indicated that COL6A3 may have been involved in the biological processes that promote the progression of GC.

### Network analysis of COL6A3

**Figure 3:**
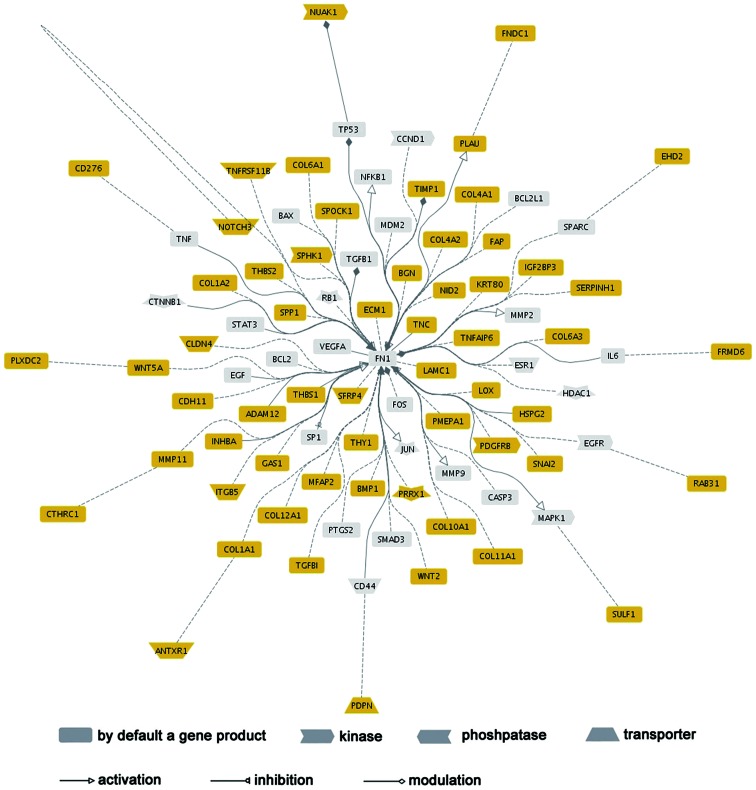
Network construction of COL6A3 co-expressed genes. The biological interactions of COL6A3 co-expressed genes were analyzed and visualized using a gene expression pattern scanner. The category of each gene is distinguished by its shape for factors, such as kinases and transporters. The direction of the arrow demonstrates whether a gene is upstream or downstream of another gene. Dashed line, co-cited genes; solid line, genes with an expertly curated connection. Genes with no interactions are not shown.

A network analysis was conducted using Genomatix GePS to construct the functional connections of COL6A3 co-expressed genes. FN1 was highlighted in this network, as it functionally associated with 50 (81.9%) COL6A3 co-expressed genes, which indicated that FN1 may act as a significant regulator in the COL6A33 regulatory network (Fig. 3).

## Discussion

COL6A3 is located on chromosome 2q37 and codes for the α-3 chain, one of the three α-chains of type VI collagen. It is hypothesized that COL6A3 accelerates cell anchoring and signaling through its interaction with integrin (17) and disruption of this gene results in muscular dystrophy (2). In addition to integrin, COL6A3 interacts with other matrix components, such as decorin, hyaluronan, heparan sulfate and NG2 proteoglycans (18). Furthermore, COL6A3 may promote neural crest cell migration and attachment, which is significant in the later stages of neural crest development (19).

Recently, COL6A3 has received increasing attention, due to its abnormal expression and the occurrence of alternative splicing in numerous types of cancer. Previous genome exon array studies have identified cancer-specific alternative splicing of exons 3, 4 and 6 of COL6A3 in colon, pancreatic, bladder and prostate cancer (8,20). Furthermore, COL6A3 was identified to be overexpressed in pancreatic (21) and ovarian cancer (7), which was associated with the poor differentiation of tumor cells (5). Although COL6A3 has been investigated in numerous other types of cancer, its biological mechanisms and expression pattern in GC remain unclear.

In the era of post-genomic medicine, microarray meta-analysis has been demonstrated to be an effective strategy for identifying gene expression changes in various types of cancer (22,23). In the present study, a microarray meta-analysis was performed to identify that COL6A3 was frequently overexpressed in hepatocellular carcinoma tissues, indicating that an increased expression of COL6A3 was associated with the carcinogenesis of GC. The underlying mechanisms that result in the increased expression of COL6A3 may relate to the transcriptional regulation of transforming growth factor (TGF)-β (24), however, this requires further investigation. To further define the biological mechanisms of COL6A3, a co-expression analysis was conducted to investigate the genes that are functionally related to, or co-regulated by, COL6A3. This identified 62 co-expression genes for COL6A3, the majority of which are involved in the processes of extracellular matrix organization such as lysyl oxidase, collagen type IV α2, TGF-β-induced and laminin γ1 (Table II). The functional network analysis of these co-expression genes was dominated by FN1, which demonstrated its predominant functional connections with other genes. FN1 is an adhesive protein of the extracellular matrix and it contains two apparently identical subunits with a range of binding sites for cell surface and extracellular ligands. It has been indicated that FN1 is involved in various aspects of cancer-related biological processes, such as cellular adhesion and migration. FN1 was identified to be overexpressed in hepatocellular, gastrointestinal, head and neck cancers (25,26), which indicated its involvement in tumorigenesis. Furthermore, Waalkes demonstrated that advanced-stage renal cancer patients exhibited increased FN1 expression when compared with patients exhibiting organ-confined diseases (27). Thus, the present study provided a mechanistic insight into the role of COL6A3 in GC.

In conclusion, the present study indicated that COL6A3 was regularly overexpressed in GC cells. A list of potential partner genes of COL6A3 was generated, the majority of which are involved in cancer-related processes, and a functional network of COL6A3 was constructed, which provided promising results to enable future studies to identify the precise role of COL6A3.

## Figures and Tables

**Table I:** Oncomine datasets obtained for use in the present study.

Dataset (Ref no.)	Samples	Data link
Chen Gastric (11)	103 gastric adenocarcinomas and 29 normal gastric mucosa samples	http://genome-www.stanford.edu/gastric_cancer2/index.shtml
Cho Gastric (12)	65 gastric adenocarcinoma, 19 paired surrounding normal tissue and six gastrointestinal stromal tumor samples	http://www.ncbi.nlm.nih.gov/geo/query/acc.cgi?acc=GSE13861
D’Errico Gastric (13)	31 paired gastric carcinoma and adjacent normal gastric mucosa and seven unmatched gastric carcinoma samples	http://www.ncbi.nlm.nih.gov/geo/query/acc.cgi?acc=GSE13911
Wang Gastric (14)	12 paired gastric carcinoma and normal gastric mucosa samples and three normal gastric tissue samples	http://www.ncbi.nlm.nih.gov/geo/query/acc.cgi?acc=GSE19826

**Table II:** Collagen VI α3 co-expressed genes with the cut-off for selection defined as an appearance in two datasets.

Gene	Gene name	No. of appearances
COL6A3	Collagen type VI α3	3
COL1A2	Collagen type I α2	3
COL1A1	Collagen type I α1	3
COL12A1	Collagen type XII α1	3
THY1	Thy-1 cell surface antigen	3
THBS2	Thrombospondin 2	3
BGN	Biglycan	3
CTHRC1	Collagen triple helix repeat containing 1	3
SULF1	Sulfatase 1	3
FAP	Fibroblast activation protein-α	3
SFRP4	Secreted frizzled-related protein 4	3
TIMP1	Tissue inhibitor of metallopeptidase 1	3
WNT2	Wingless-type mouse mammary tumor virus integration site family member 2	3
COL11A1	Collagen type XI α1	3
BMP1	Bone morphogenetic protein 1	3
SPOCK1	Sparc/osteonectin cwcv and kazal-like domains proteoglycan (testican) 1	3
SERPINH1	Serpin peptidase inhibitor clade H (heat shock protein 47) member 1 (collagen binding protein 1)	2
CPXM1	Carboxypeptidase X (M14 family) member 1	2
INHBA	Inhibin β A	2
CDH11	Cadherin 11, type 2, OB-cadherin (osteoblast)	2
RAB31	Member of the RAS oncogene family	2
ANTXR1	Anthrax toxin receptor 1	2
NID2	Nidogen 2 (osteonidogen)	2
PDGFRB	Platelet-derived growth factor receptor β polypeptide	2
COL4A2	Collagen type IV α2	2
COL4A1	Collagen type IV α1	2
TGFBI	Transforming growth factor β-induced (68kDa)	2
PLAU	Plasminogen activator urokinase	2
PRRX1	Paired related homeobox 1	2
LOX	Lysyl oxidase	2
PLXDC2	Plexin domain containing 2	2
LAMC1	Laminin γ1 (formerly LAMB2)	2
OLFML2B	Olfactomedin-like 2B	2
CLDN4	Claudin 4	2
FAM83D	Family with sequence similarity 83, member D	2
ITGB5	Integrin β5	2
TNC	Tenascin C	2
SNAI2	Snail family zinc finger 2	2
FRMD6	FERM domain containing 6	2
COL6A1	Collagen type VI α1	2
NUAK1	NUAK family, SNF1-like kinase 1	2
HSPG2	Heparan sulfate proteoglycan 2	2
NOTCH3	Notch 3	2
CD276	Cluster of differentiation 276 molecule	2
WNT5A	Wingless-type mouse mammary tumor virus integration site family member 5A	2
ECM1	Extracellular matrix protein 1	2
PDPN	Podoplanin	2
TNFAIP6	Tumor necrosis factor α-induced protein 6	2
ADAM12	A disintegrin and metallo-peptidase domain 12	2
GAS1	Growth arrest-specific 1	2
THBS1	Thrombospondin 1	2
COL10A1	Collagen type X α1	2
FNDC1	Fibronectin type III domain containing 1	2
SPHK1	Sphingosine kinase 1	2
MMP11	Matrix metallopeptidase 11 (stromelysin 3)	2
CST1	Cystatin SN	2
KRT80	Keratin 80	2
PMEPA1	Prostate transmembrane protein, androgen induced 1	2
SPP1	Secreted phosphoprotein 1	2
TNFRSF11B	Tumor necrosis factor receptor superfamily, member 11b	2
IGF2BP3	Insulin-like growth factor 2 mRNA binding protein 3	2
MFAP2	Microfibrillar-associated protein 2	2
EHD2	EH-domain containing 2	2

**Table III:** GO and pathway enrichment analysis of COL6A3 co-expressed genes.

Category	Term	Function	Count	P-value	Fold enrichment	FDR
GOTERM _BP_3	GO:0030198	ECM organization	11	5.88×10^−12^	26.86738026	8.02×10^−9^
	GO:0048731	System development	29	2.30×10^−9^	3.161608227	3.13×10^−6^
	GO:0048513	Organ development	24	2.54×10^−8^	3.507740409	3.47×10^−5^
	GO:0009653	Anatomical structure morphogenesis	19	2.42×10^−7^	4.032045523	3.30×10^−4^
	GO:0009888	Tissue development	14	9.88×10^−7^	5.347765641	1.35×10^−3^
	GO:0022603	Regulation of anatomical structure morphogenesis	7	1.89×10^−4^	8.119324546	2.57×10^−1^
	GO:0030154	Cell differentiation	17	3.01×10^−4^	2.637947926	4.10×10^−1^
	GO:0051093	Negative regulation of developmental process	7	4.64×10^−4^	6.865374809	6.31×10^−1^
	GO:0031589	Cell-substrate adhesion	5	5.47×10^−4^	12.96014632	7.43×10^−1^
	GO:0051239	Regulation of multicellular organismal process	12	7.52×^−4^	3.253176537	1.02
	GO:0048519	Negative regulation of biological process	17	9.44×10^−4^	2.383179224	1.28
	GO:0050793	Regulation of developmental process	10	9.95×10^−4^	3.768825934	1.35
	GO:0060348	Bone development	5	1.28×10^−3^	10.32597024	1.73
	GO:0009887	Organ morphogenesis	9	1.33×10^−3^	4.053492573	1.80
	GO:0006928	Cell motion	8	2.20×10^−3^	4.278212512	2.96
	GO:0042127	Regulation of cell proliferation	10	2.90×10^−3^	3.227685742	3.88
	GO:0032101	Regulation of response to external stimulus	5	3.27×10^−3^	7.988014715	4.36
	GO:0002683	Negative regulation of immune system process	4	4.02×10^−3^	12.24187315	5.34
	GO:0009611	Response to wounding	8	4.05×10^−3^	3.834247063	5.39
	GO:0030334	Regulation of cell migration	5	4.06×10^−3^	7.515351122	5.40
	GO:0016477	Cell migration	6	4.13×10^−3^	5.522149303	5.49
	GO:0016337	Cell-cell adhesion	6	4.13×10^−3^	5.522149303	5.49
	GO:0050865	Regulation of cell activation	5	4.60×10^−3^	7.257681941	6.08
	GO:0008284	Positive regulation of cell proliferation	7	5.02×10^−3^	4.295005013	6.64
	GO:0009790	Embryonic development	8	5.90×10^−3^	3.577730534	7.75
	GO:0007566	Embryo implantation	3	5.94×10^−3^	25.40188679	7.80
	GO:0044259	Multicellular organismal macromolecule metabolic process	3	6.33×10^−3^	24.58247109	8.30
	GO:0040012	Regulation of locomotion	5	6.37×10^−3^	6.615074686	8.34
	GO:0051272	Positive regulation of cell motion	4	6.39×10^−3^	10.36811706	8.36
	GO:0040017	Positive regulation of locomotion	4	6.39×10^−3^	10.36811706	8.36
	GO:0048870	Cell motility	6	6.46×10^−3^	4.964538135	8.46
	GO:0051270	Regulation of cell motion	5	6.48×10^−3^	6.580799687	8.49
	GO:0048523	Negative regulation of cellular process	14	8.98×10^−3^	2.142327802	11.57
	GO:0050867	Positive regulation of cell activation	4	9.00×10^−3^	9.153833078	11.59
	GO:0009792	Embryonic development ending in birth or egg hatching	6	9.13×10^−3^	4.563213196	11.76
	GO:0032844	Regulation of homeostatic process	4	9.67×10^−3^	8.912942734	12.41
GOTERM_CC_3	GO:0031012	ECM	26	2.51×10^−26^	19.52139523	2.54×10^−23^
	GO:0005578	Proteinaceous ECM	25	1.40×10^−25^	20.23702331	1.41×10^−22^
	GO:0044420	ECM part	15	8.00×10^−18^	33.20947414	8.10×10^−15^
	GO:0005581	Collagen	10	5.53×10^−15^	74.00968523	5.62×10^−12^
	GO:0005604	Basement membrane	6	1.13×10^−5^	19.92568449	1.14×10^−2^
	GO:0005615	Extracellular space	12	4.49×10^−5^	4.537820116	4.55×10^−2^
	GO:0005886	Plasma membrane	25	3.82×10^−3^	1.71454791	3.80
	GO:0031252	Cell leading edge	3	9.65×10^−2^	5.631171702	64.22
GOTERM_MF_3	GO:0019838	Growth factor binding	6	3.98×10^−5^	15.21982507	3.80×10^−2^
	GO:0005518	Collagen binding	4	3.06×10^−4^	29.59410431	2.92×10^−2^
	GO:0005102	Receptor binding	11	1.21×10^−3^	3.306790436	1.15
KEGG_PATHWAY	hsa04512	ECM-receptor interaction	14	1.41×10^−16^	28.25	9.99×10^−14^
	hsa04510	Focal adhesion	14	1.52×10^−11^	11.80597015	1.35×10^−08^

GO, gene ontolgy; COL6A3, collagen VI α3; FDR, false discovery rate; BP; biological process; CC, cellular constituent; ECM, extracellular matrix; MF, molecular function; KEGG, Kyoto encyclopedia of genes and genomes. P<0.01 indicated a statistically significant difference.
